# The correlation between periodontitis and uncontrolled hypertension is mediated by inflammatory markers: results from a cross-sectional study of urban elderly population in southeast China

**DOI:** 10.1186/s12903-023-03680-x

**Published:** 2023-11-24

**Authors:** Yue Chen, Jiaoer Zheng, Da Ni, Denghui Zhang, Haihua Zhu

**Affiliations:** Stomatology Hospital, School of Stomatology, Zhejiang University School of Medicine, Zhejiang Provincial Clinical Research Center for Oral Diseases, Key Laboratory of Oral Biomedical Research of Zhejiang Province, Cancer Center of Zhejiang University, Hangzhou, 310006 Zhejiang China

**Keywords:** Hypertension, Blood pressure, Uncontrolled, Periodontitis, Inflammation

## Abstract

**Background:**

Periodontal diseases is considered the most important global oral health burden according to the world health organization (WHO) (Oral health. https://www.who.int/news-room/fact-sheets/detail/oral-health#Overviewth (who.int). Accessed 21 Sep 2023). It is a common local inflammatory disease associated with hypertension, this study aims to explore the relationship between periodontitis and uncontrolled hypertension and whether inflammation indication such as white blood cell (WBC) count or neutrophil count is a mediator of this relationship.

**Methods:**

One thousand four hundred eighty-eight elders attending annual physical and oral examinations in Zhejiang province were included in this study. The staging of Periodontitis was classified as none, mild-moderate and severe. Participants are categorized into two groups based on blood pressure: hypertensive( positive high blood pressure( HBP) history or underwent HBP medication or blood pressure( BP) ≥ 140/90 mmHg) and uncontrolled hypertensive (systolic blood pressure( SBP) ≥ 140 mmHg or distolic blood pressure( DBP) ≥ 90 mmHg). Peripheral blood samples were collected, information on hypertension history and potential confounders (age, sex, smoking, alcohol consumption, exercise frequency, diabetes) was collected in questionnaires. The correlation between periodontitis and hypertension was investigated using logistics regression analyses, mediation analysis was assessed for the effect of inflammation on hypertension.

**Results:**

The study population includes 1,488 participants aged 55–90 years. Odds of uncontrolled hypertension increased significantly along with periodontitis in the regression models both in unadjusted model (odds ratio( OR): 1.407, 95% confidence intervals( CI): 1.037 ~ 1.910) and fully adjusted model (OR: 1.950, 95% CI: 1.127 ~ 3.373). Mediation analysis confirmed that WBC and neutrophic count function as a full mediator of the association between periodontitis and uncontrolled hypertension either in the unadjusted or the adjusted model.

**Conclusions:**

In a study of urban elderly population in southeast China, periodontitis is found to be significantly associated with uncontrolled hypertension, such relation is mediated by WBC and neutrophil count. Periodontitis can increase the difficulty of controlling hypertension. Promotion of periodontal health strategies in the dental setting could help reduce the burden of hypertension and its complications.

## Background

Periodontal disease is one of the most frequent diseases with a prevalence of 45–50% of the world population [1], almost 750 million people aged from 15 to 99 worldwide present with moderate to severe symptoms of periodontitis [2]. The Chinese fourth national oral health survey revealed that the prevalence of periodontitis in people over 55 years old is 64.6%, and the prevalence of severe periodontitis is 37.3% [3]. Periodontal disease is associated with many non-communicable diseases( NCDs) including cardiovasculardisease [4], diabetes [5], chronic respiratory disease [6], obesity [7] and chronic kidney disease [8]. If not treated effectively, the disease ultimately leads to tooth loss, decreased oral function and negatively affects patients’ quality of life [9, 10].

Systemic arterial hypertension is the most prevalent chronic NCD in the world. Numerous epidemiological studies have demonstrated a strong association between hypertension and the development of various cardiovascular diseases, including coronary artery disease, heart failure, stroke, and peripheral vascular disease. The population attributable fraction of high blood pressure for cardiovascular disease mortality is 40.6% (95% CI, 24.5%–54.6%) [11–13]. More than 30% of adults are estimated to have the disease worldwide with higher frequencies found with increasing age [14]. A 15% to 50% of individuals, however, are unaware of their hypertension, [15] meanwhile many of those with an established diagnosis fail to achieve an optimal BP control despite their prescribed medications [11].

Previous epidemiological studies and meta-analyses have reported the relation between oral health and cardiovascular diseases, suggesting periodontal disease is associated with hypertension [15–20]. However the relationship and mediation between periodontitis and uncontrolled hypertension has not been reported. Since hypertension and its control level is closely linked with socioeconomic status (SES), and Hangzhou is holding the 19th Asian Games in Sep. 2023, which represents the economically developed regions of China. Data on China’s economically developed regions is inadequate, we performed this cross-sectional study to investigate the relationship between the two diseases in Hangzhou and find out the mediation of the relationship.

## Materials and methods

### Population and study design

We used data on urban elderly population (aged from 55–90 years) from a routine physical examination from 6 community hospitals in Hangzhou, capital city of Zhejiang province which has a high economic level according to the national gross domestic product in mainland China. This study was conducted following the Declaration of Helsinki and approved by the Ethics Committee of Stomatology Hospital Affiliated to Zhejiang University School of Medicine (Ethics Approval No. 2021–075).

Exclusion criteria for study participants were as follows: (1) data for blood test or questionnaires were missing, (2) did not complete oral and periodontal examinations. (3) presence of systemic infections, (4) systemic antibiotic treatment in the past 3 months, (5) undergoing periodontal treatment in the past three months.

Five thousand eight hundred nineteen participants were attending the routine physical examination in 2021, of which 4014 received oral examinations and blood pressure values were available for 3668 participants. After excluding those who did not perform oral and systemic examinations, 1488 participants remained.

A post hoc power analysis was carried out using the G-POWER 3.1 programme at the end of the study. At an effect size of 0.02 and α of 0.05, a sample size of 1400 can achieve a power of 0.97.

### Periodontal examination and diagnosis

All the periodontal examinations were finished by well-trained and calibrated periodontists. Periodontal examinations were conducted on two-diagonal quadrants randomly, one in the maxilla and the other in the mandible, excluding third molars and implants. Measurements were taken at four sites per tooth (distobuccal (DB), mesiobuccal (MB), mesiolingual (ML), and distolingual (DL)). Probing depth (PD) and clinical attachment loss (CAL) were assessed by a manual UNC-15 probe (Hu-Friedy, Chicago, IL). Tooth loss was also recorded at the same time. Periodontitis status was classified into three categories, namely, severe periodontitis, mild-moderate periodontitis, and no periodontitis. Periodontitis classification was based on a modified half-reduced CDC/AAP definition (CDC, Center of Disease Control; AAP, the American Academy of Periodontology) [21, 22]. Severe periodontitis was defined as ≥ 1 interproximal site with ≥ 6 mm CAL and ≥ 1 interproximal site(s) with ≥ 5 mm PD (on the same site with CAL ≥ 6 mm or different sites). Moderate periodontitis was defined as ≥ 1 interproximal site with ≥ 4 mm CAL or 1 site with ≥ 5 mm PD. Mild periodontitis was defined as ≥ 1 interproximal site with CAL ≥ 3 mm, and ≥ 1 interproximal sites with PD ≥ 4 mm (on the same site with CAL ≥ 3 mm or different sites). No periodontitis was defined as no evidence of mild, moderate, or severe periodontitis [23].

### Assessment of arterial hypertension

The diagnosis of arterial hypertension was based on a medical history, with focus on antihypertensive drugs, a blood pressure measurement, and patients’ self-report. Blood pressure was measured on both arms each for 2 times after the initial rest of 5 min and a one-minute interval between each. Resting BP was recorded and calculated as the average of 4 readings. The Sixth Report of the Joint National Committee on Prevention, Detection, Evaluation, and Treatment of High Blood Pressure defined that the diagnosis of hypertension is made when the average of 2 or more diastolic BP measurements on at least 2 subsequent visits is ≥ 90 mm Hg or when the average of multiple systolic BP readings on 2 or more subsequent visits is consistently ≥ 140 mm Hg [24]. Hypertensive individuals were classified into 2 groups:” hypertensive”: with a positive HBP history or underwent HBP medication or BP ≥ 140/90 mmHg, “uncontrolled hypertension”: participants who fail to maintain to less than 140/90 mm Hg regardless of medication use or treatment [11, 25].

### Confounders

Questionnaires were distributed to all participants, including sex, age, height, weight, alcohol consumption, exercise frequency, and smoking habits. sex was categorized as female and male. Body mass index (BMI) was calculated from weight /height2 (kg/m2). Alcohol consumption was classified into 3 groups: (1) never, (2) often, and (3) daily. Exercise frequency was categorized into 3 groups: (1) never, (2) 1–3 times per week, (3) 4–7 times per week [26]. Smoking habits were classified into 3 groups: (1) current smoker, (2) former smoker, and (3) nonsmoker. Former smokers were defined as those who had smoked in the past but quit smoking for at least 6 months. WBC, neutrophil count, lymphocyte count, Fasting glucose and triglycerides, high-density lipoprotein( HDL); low-density lipoprotein( LDL), and total cholesterol were measured by routine laboratory tests.

### Statistical analysis

Data analysis was performed using IBM SPSS Statistics version 25.0 for Windows (IBM, ARMONK, NY, USA). Continuous variables were reported as means ± standard deviation (SD). Categorical variables were reported as number of cases (n) and percentage (%). Comparisons between different periodontal status were conducted using chi-squared test for categorical variables and ANOVA for continuous ones.

We employed logistic regression models to evaluate the multivariable association between periodontal disease and hypertension. Independent variables were selected among clinical and demographic characteristics, age, sex, systolic blood pressure, diastolic blood pressure, fasting plasma glucose, cholesterol, triglycerides, high-density lipoprotein, white blood cells, neutrophil count, lymphocyte count, body mass index, smoking and alcohol consumption were included. Following the initial crude model (model 1, unadjusted), 4 extended models were generated with adjustment for age and sex (model 2), additional inclusion of BMI and waist circumference (model 3), an additional inclusion of inflammation indexes including white blood cell, neutrophil count, and lymphocyte count (model 4), lifestyle habits including smoking, exercising and alcohol consumption were added in model 5. Odds ratios and 95% CI were reported.

Finally, we used 2 mediation models to identify and explain the pathways or processes underlying the relationship between periodontitis and uncontrolled hypertension, via the hypothetical mediator of inflammation factors.

## Results

### Baseline characteristics of the study group

Baseline characteristics of the study are presented in Table 1, subdivided by the severity of periodontitis. Our final sample after subject selection includes 1,488 participants aged 55–90 years, of which 602 (40.46%) were male and 886 (59.54%) were female. Their mean age was 64.446 ± 7.765 years. 1254(84.27%) had a history of hypertension diagnosis or were observed to have high blood pressure, 924(62.10%) were observed to have high blood pressure, the prevalence of uncontrolled hypertension was 63.46% (382/602) for men and 61.17% (542/886) for women. The prevalence of periodontal disease was 1295(87.03%), including 539(36.22%) people exhibited severe periodontitis and 756(50.81%) people exhibited mild-moderate periodontitis and 193(12.97%) exhibited no periodontitis. Of those who had periodontitis the prevalence for male is 91.03% (548/602) and 84.31% (747/886) for female.

**Table 1:** Characteristics of the study population according to periodontal status (*n* = 1488)

		Staging of periodontitis (%)			Total (*n* = 1488)	*p*
		None (*n* = 193)	Mild-moderate (*n* = 756)	Severe (*n* = 539)		
Age, y		63.20 ± 7.39	64.44 ± 8.06	64.90 ± 7.43	64.44	0.034*
sex, n (%)	Male	54 (27.98)	293 (38.76)	255 (47.31)	602 (40.46)	0.000**
	Female	139 (72.02)	463 (61.24)	284 (52.69)	886 (59.54)	
Hypertension, n (%)	No	35 (18.13)	117 (15.48)	82 (15.21)	234 (15.73)	0.610
	Yes	158 (81.87)	639 (84.52)	457 (84.79)	1254 (84.27)	
Uncontrolled Hypertension, n (%)	No	87 (45.08)	271 (35.85)	206 (38.22)	564 (37.90)	0.061
	Yes	106 (54.92)	485 (64.15)	333 (61.78)	924 (62.10)	
Alcohol consumption, n (%)	Never	50 (75.76)	211 (69.64)	156 (67.83)	417 (69.62)	0.631
	Heavy	14 (21.21)	87 (28.71)	70 (30.43)	171 (28.55)	
	Binge	2 (3.03)	5 (1.65)	4 (1.74)	11 (1.84)	
Exercise frequency, n (%)	Never	16 (24.24)	94 (31.02)	63 (27.39)	173 (28.88)	0.217
	1-3times/week	20 (30.30)	55 (18.15)	45 (19.57)	120 (20.03)	
	4–7 times/week	30 (45.45)	154 (50.83)	122 (53.04)	306 (51.09)	
Smoking Habits, n (%)	Never	171 (92.43)	687 (91.72)	491 (91.09)	1349 (91.58)	0.516
	Current	9 (4.86)	49 (6.54)	41 (7.61)	99 (6.72)	
	Former	5 (2.70)	13 (1.74)	7 (1.30)	25 (1.70)	
SBP, mmHg		141.67 ± 18.59	143.67 ± 17.46	143.02 ± 17.96		0.364
DBP, mmHg		83.83 ± 9.73	83.52 ± 10.59	83.45 ± 10.63		0.907
Fpg, mmol/L		6.14 ± 1.39	6.39 ± 1.65	6.56 ± 1.87		0.011*
lymphocyte percentage (%)		35.42 ± 7.92	34.68 ± 7.78	33.81 ± 7.73		0.026*
Neutrophils, 10^9/L		3.59 ± 1.20	3.70 ± 1.20	3.88 ± 1.35		0.006**
Monocyte, 10^9/L		0.36 ± 0.12	0.38 ± 0.12	0.39 ± 0.13		0.006**
WBC, 10^9/L		6.33 ± 1.60	6.48 ± 1.61	6.70 ± 1.75		0.011*
total cholesterol, mmol/L		5.21 ± 1.07	5.23 ± 1.03	5.15 ± 1.05		0.369
triglycerides, mmol/L		1.57 ± 0.98	1.62 ± 0.98	1.67 ± 1.05		0.413
HDL, mmol/L		1.47 ± 0.36	1.44 ± 0.36	1.39 ± 0.35		0.005**
BMI, kg/m^2^		26.70 ± 32.54	24.20 ± 3.21	24.73 ± 3.11		0.043*

*SBP* systolic blood pressure, *DBP* Diastolic Blood Pressure, *FPG* Fasting plasma glucose, *WBC* white blood cells, *HDL* high-density lipoprotein, *BMI* indicates body mass index

* *p* < 0.05 ** *p* < 0.01

### Association between periodontitis and hypertension

Of the hypertensive participants (with both hypertension history and a tested high blood pressure), after adjusting for all potential confounders, we did not observe any association between periodontitis and hypertension. When we categorized our study population into periodontally healthy and periodontitis and restrict our analysis to UNCONTROLLED high blood pressure only, we found a significant effect of periodontitis on the odds of hypertension (Table 2). In the unadjusted model (model1), odds ratio (OR) for uncontrolled hypertension is 1.407, 95% confidence interval (CI) is 1.037 ~ 1.910, *p* < 0.05. After adjusted for sex and age (model2), OR and 95% CI is 1.367(1.005 ~ 1.861), *p* < 0.05. Model3 introduced serum lipids indexes, OR and 95% CI is 1.405(1.024 ~ 1.928), *p* < 0.05. Model4 introduced inflammation indexes, OR and 95% CI is 1.421(1.034 ~ 1.953), *p* < 0.05. Model5 adjusted for lifestyle habits, OR and 95% CI gets to 1.950(1.127 ~ 3.373), *p* < 0.05. The result of the regression models confirmed that the parameter selection of the periodontitis models does not affect the result of the study that odds of uncontrolled hypertension increased significantly along with periodontitis in the regression models.

**Table 2:** Odds ratios (OR) and correspondent 95% confidence intervals (CI) towards uncontrolled hypertension, according to the periodontal status, calculated within logistic regression analyses for different adjustment levels

Model	p	OR	95% CI
Model1	0.028*	1.407	1.037 ~ 1.910
Model2	0.047*	1.367	1.005 ~ 1.861
Model3	0.035*	1.405	1.024 ~ 1.928
Model4	0.03*	1.421	1.034 ~ 1.953
Model5	0.017*	1.95	1.127 ~ 3.373

Model1: unadjusted model; Model2: adjusted for sex and age based on model1; Model3: adjusted for BMI, waist circumference, TC, TG, HDL, LDL, FBG based on model2; Model4:adjusted for inflammation indexes based on model3; Model5:adjusted for smoking habits, alcohol assumption, exercising frequency based on model4

* *p* < 0.05

### Mediation analysis

Because both a (the regression coefficient of periodontitis on mediators) and b (the regression coefficient of mediators on uncontrolled hypertension) are significant, and c' (the regression coefficient of periodontitis on uncontrolled hypertension) is not significant, it is considered that WBC and neutrophil count function as a full mediator of the association between periodontitis and uncontrolled hypertension either in the unadjusted or the fully adjusted model. While lymphocyte count did not function as a mediator. Results of the mediation analysis are summarized in Table 3.

**Table 3:** Mediation analysis: periodontitis (exposure), uncontrolled hypertension (outcome), inflammation indexes (mediator)

	route	c total effect	a	b	a*b	a*b (95% BootCI)	c’	conclusion
model1	periodontitis = > WBC = > uncontrolled hypertension	0.019	0.195**	-0.172**	-0.034	-0.103 ~ -0.009	0.02	full mediation
	periodontitis = > neutrophil count = > uncontrolled hypertension	0.019	0.155**	0.192**	0.03	0.009 ~ 0.089	0.02	full mediation
	periodontitis = > lymphcyte count = > uncontrolled hypertension	0.019	0.012	0.205**	0.002	-0.014 ~ 0.021	0.02	not significant
model2	periodontitis = > WBC = > uncontrolled hypertension	0.015	0.149*	-0.174**	-0.034	-0.102 ~ -0.001	0.017	full mediation
	periodontitis = > neutrophil count = > uncontrolled hypertension	0.015	0.136*	0.104**	0.031	0.001 ~ 0.091	0.017	full mediation
	periodontitis = > lymphcyte count = > uncontrolled hypertension	0.015	0.013	0.266**	0.009	-0.013 ~ 0.018	0.017	not significant

Model 1: Unadjusted model

Model 2: Adjusted for Age, sex, BMI, HDL, alcohol consumption( never, heavy, binge), exercise frequency( never, 1-3times/week, 4-7times/week), and smoking habits( never, current, former)

c: the regression coefficient of PD on uncontrolled hypertension (when there is no mediator variable in the model), which is the total effect; a: the regression coefficient of exposure (PD) on mediators; b: the regression coefficient of mediators on uncontrolled hypertension; a*b: the indirect effect; c': the regression coefficient of PD on uncontrolled hypertension (when there is a mediator variable in the model), which is the direct effect

* *p* < 0.05 ** *p* < 0.01

## Discussion

Currently, there is a lack of standardized diagnostic criteria for periodontitis in epidemiological surveys, making Full mouth periodontal examinations (FMPE) the “gold standard” for evaluating periodontal health. In our study, we implemented a protocol for partial mouth periodontal examinations (PMPE). Since PMPE protocols involve examining fewer probing sites compared to FMPE, applying a case definition designed for FMPE may result in underestimating the prevalence of the disease. Among various protocols for PMPE, such as Ramfjord teeth (RT) and community periodontal index (CPI), the two random diagonal quadrants protocol demonstrates the highest level of accuracy [27]. For our study, we utilized a modified half-reduced CDC/AAP definition that was specifically tailored for PMPE [22]. It has been reported that when using the half-reduced CDC/AAP definition, the half-mouth four-site protocol yielded minimal absolute bias in estimating the prevalence of moderate periodontitis and severe periodontitis compared to FMPE.

According to our study findings, the presence of periodontitis was associated with elevated blood pressure. However, there was no statistically significant difference between the severity of periodontitis and either SBP or DBP, nor with the prevalence of hypertension. Similar findings have been observed in other observational studies conducted among various populations, including Korean female adults [16], the Puerto Rico elderly population [17], the central China population [18], the hamburg population [19], and the Portuguese population [20]. These studies have also reported a significant positive correlation between periodontal disease and an increased risk of hypertension. However, the factors contributing to this correlation are complex and not fully understood. Systemic inflammation and subsequent vascular endothelial damage are considered the primary pathomechanisms responsible for the elevation of blood pressure in individuals with periodontitis [28–30]. Periodontitis triggers a systemic inflammatory response mediated by various factors, including C-reactive protein, interleukin 1b (IL-1b), interleukin 6 (IL-6), tumor necrosis factor alpha (TNF-α), and others [31]. These factors can directly impact the vascular endothelium, resulting in impaired vasodilatory function [32]. The findings of the genome-wide association study revealed an association between single nucleotide polymorphisms (SNPs) and periodontitis [33]. A study by Czesnikiewicz-Guzik et al. showed that all four studied SNPs showed also concordant effect direction [34]. The outcomes of this study provide an explanation for one potential reason behind the frequent coexistence of both periodontitis and arterial hypertension.

There are some limitations in the study. As our oral epldemiological study was carried out on a community level, the x-ray were not taken and radiographic bone loss could not be assesed. Secondly, the study categorized hypertensive patients into two groups: hypertension vs. uncontrolled hypertension. It could be more accurate if reclassified as controlled hypertension (defined as having a medical history or currently taking medication with blood pressure under control) vs. uncontrolled hypertension. However, due to medical history being based on self-reported questionnaires, its accuracy is insufficient. Also, lack of case–control studies is a limitation of cross-sectional research, and blood pressure outcomes following periodontal treatment were not provided. Further research is needed to enhance the evidence supporting this correlation and to elucidate the mechanisms through which periodontal pathogens or the subsequent inflammation contribute to elevated blood pressure.

China has become an aging society due to improvement in living standards and the extension of life expectancy. Our study took place in Hangzhou, capital city of Zhejiang province. It’s holding the 19th Asian Games in Sep. 2023, which represents the economically developed regions of China, thus the data reflects a general trend for the impact periodontal status puts on general health. The National Healthcare Security Administration of China is reducing oral medical cost though centralized procurement, considering oral health is an important factor that affects the quality of life, policymakers need to address oral health needs and consider adding coverage for comprehensive oral health benefits.

## Conclusion

The study found the significant association between periodontitis and uncontrolled hypertension is mediated by WBC and neutrophil count. Periodontitis can increase the difficulty of controlling hypertension. Promotion of periodontal health strategies in the dental setting could help reduce the burden of hypertension and its complications.

## Data Availability

The data that support the findings of this study are available from the corresponding author, but restrictions apply to the availability of these data, which were used under license for the current study, and so are not publicly available. Data are however available from the authors upon reasonable request and with permission of the corresponding author.

## Abbreviations

WHO: World Health Organization
WBC: White blood cells
HBP: High blood pressure
BP: Blood pressure
SBP: Systolic blood pressure
DBP: Diastolic blood pressure
OR: Odds ratio
CI: Confidence interval
NCDs: Non-communicable diseases
SES: Socioeconomic status
DB: Distobuccal
MB: Mesiobuccal
ML: Mesiolingual
DL: Distolingual
PD: Probing depth
CAL: Clinical attachment loss
CDC: Center of Disease Control
AAP: The American Academy of Periodontology
BMI: Body mass index
FPG: Fasting plasma glucose
HDL: High-density lipoprotein
LDL: Low-density lipoprotein
FMPE: Full mouth periodontal examinations
PMPE: Partial mouth periodontal examination protocol
SNPs: Single nucleotide polymorphisms
